# Advances in Metal‐Free Transamidation: A Sustainable Approach to Amide Bond Formation

**DOI:** 10.1002/asia.202500327

**Published:** 2025-04-10

**Authors:** Niharan Sivaraj, Toshifumi Dohi, Fateh V. Singh

**Affiliations:** ^1^ Chemistry Division School of Advanced Sciences Vellore Institute of Technology Chennai Campus Chennai Tamil Nadu 600127 India; ^2^ College of Pharmaceutical Sciences Ritsumeikan University 1‐1‐1 Nojihigashi Kusatsu Shiga 525‐0058 Japan

**Keywords:** Amides, Catalysts, Nucleophilic, Solvent‐free, Transamidation

## Abstract

The amide functionalities are a crucial functional group in organic synthesis, playing a vital role in many processes that are essential for the efficient synthesis of important pharmaceutical and industrial compounds. Despite being one of the most commonly conducted reactions by researchers in both academia and industry, the synthesis of amides remains a staple in chemical research and development. Transamidation reactions enable the one‐pot conversion of one type of amide into another. Additionally, this process is crucial in the complete synthesis of specific naturally‐occurring compounds. However, these methods have certain limitations such as using toxic and corrosive starting materials, usage of strong acid or base, and metal mediated reaction, which can lead to excessive hydrolysis of the desired amide product. To overcome these challenges, more practical and efficient approaches have been developed. Metal‐free transamidation reactions have emerged as a powerful and versatile synthetic methodology in organic chemistry, allowing for the direct conversion of amides into new amide products without relying on metal catalysts. In the review article, we have focused on various metal‐free transamidation protocols of unactivated amides.

## Graphical abstract

This review highlights the recent developments of transamidation reactions under metal‐free reaction conditions. Various amines including weak nucleophilic amines such as aromatic amines have been used to achieve the transamidation reactions under mild and relatively green reaction conditions.

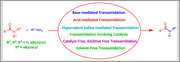

## 1 Introduction

**Figure 1:**
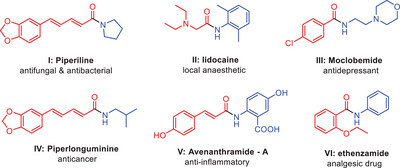
Amides present in natural products and drugs.

The amide functionalities are an essential functional group in the fields of synthetic chemistry, biology, and the production of pharmaceuticals.^[^ ^1^, ^2^, ^3^ ^]^ In the synthesis of active medicinal compounds, processes that create amide bonds are among the most routinely conducted due to the relevance of these bonds.^[^ ^4^, ^5^ ^]^ Developing new methods for amide synthesis in the chemical industry is crucial due to the significant role that amides play in both biological and synthetic polymers, natural products, synthetic intermediates, and etc.^[^ ^6^, ^7^, ^8^ ^]^ Numerous synthetic ways have been discovered for amide bond production. The most common approach is the direct reaction of a carboxylic acid with an amine, referred to as direct amidation.^[^ ^9^ ^]^ The conventional approaches to amide synthesis encounter significant obstacles, mostly due to the need to use activating agents in stoichiometric quantities, resulting in waste generation. Additionally, using caustic and troublesome reagents such as acyl chlorides or anhydrides further complicates the process.^[^ ^10^ ^]^ In recent years, numerous methods for generating amide bonds have received significant interest.^[^ ^11^ ^]^ Transamidation reactions are particularly advantageous in this situation since they provide an opportunity to expand the variety of amide bonds, which are inherent in several biologically active compounds,^[^ ^12^ ^]^ and offer distinct methods for producing a broad spectrum of beneficial amides.^[^ ^13^ ^]^ The amide system is a significant and highly favored structural motif that is extensively found in nature. Wherever the amide derivatives prominently show various biological applications such as anticancer, antimicrobial, antihypertensive activities etc. synthetically obtained drug molecules with amides functionalities such as piperiline,^[^ ^14^ ^]^ moclobemide,^[^ ^15^ ^]^ piperlonguminine,^[^ ^16^ ^]^ avenanthramide‐A,^[^ ^17^ ^]^ ethenzamide, and lidocaine.^[^ ^18^ ^]^ Transamidation has been employed in several domains, including materials research,^[^ ^19^ ^]^ polymer synthesis,^[^ ^20^ ^]^ protein engineering,^[^ ^21^ ^]^ and enzyme transformations.^[^ ^22^ ^]^ The strong stability of the N─C(O) bond, due Figure 1 to nN → π*C═O conjugation, has greatly limited the effectiveness of amides in direct transamidation reactions.^[^ ^23^ ^]^

As an outcome, numerous metal‐based catalysts for such reactions have been described.^[^ ^24^ ^]^ These include heterobimetallic complexes^[^ ^25^ ^]^ and catalysts based on scandium,^[^ ^26a^ ^]^ titanium,^[^ ^26a^ ^]^ aluminium,^[^ ^26b^ ^]^ hafnium,^[^ ^27^ ^]^ cerium,^[^ ^28^ ^]^ zirconium,^[^ ^29^ ^]^ niobium,^[^ ^30^ ^]^ nickel,^[^ ^31^ ^]^ copper,^[^ ^32^ ^]^ iron,^[^ ^33^ ^]^ and palladium.^[^ ^34^ ^]^ Nevertheless, several of these approaches have disadvantages, such the need for expensive work‐up processes, high temperatures, or catalysts composed of valuable metals. In recent years, there has been growing interest in developing metal‐free transamidation methods, which offer several advantages over metal‐mediated processes. A metal‐free approach is less expensive and less harmful to the environment. They also simplify reaction conditions, which increases their accessibility and safety for a wider variety of applications. In this detailed review, we have collaboratively examined the latest studies on metal‐free transamidation reactions.

## 2 Metal‐Free Catalyzed Transamidation Reactions

### 2.1 Base‐Mediated Transamidation Reactions

**Scheme 1:**
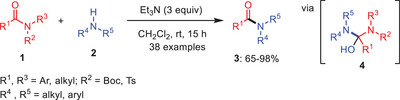
Metal‐free transamidation of *N*‐activated secondary amides **1** under neat condition.

In 2017, Liu et al. introduced a metal‐free transamidation method for *N*‐activated secondary amides **1** under mild conditions (Scheme 1). This approach relies on selectively activating amides bearing *N*‐Boc or *N*‐Ts groups, which destabilize the amide both electronically and structurally. Subsequent nucleophilic addition of amines **2** leads to tetrahedral intermediates **4** that collapse thermodynamically to produce transamidation products **3**. The efficiency of this process depends on the electronic properties of the Boc or Ts leaving groups. This adaptable method accommodates various amides and nucleophilic amines, enabling a one‐pot *N*‐activation/transamidation sequence, making it a practical and selective strategy for amide bond transformations.^[^ ^35^ ^]^

**Scheme 2:**
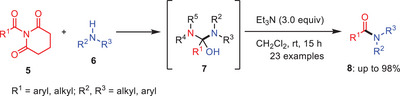
*N*‐Acyl‐glutarimide **5** transamidation with amines **6** under mild reaction conditions.

Later in 2018, the same group utilize the same conditions to transamidate *N*‐acyl‐glutarimides **5** with amines **6** (Scheme 2). When compared to conventional approaches, this novel strategy not only increases the transamidation process's efficiency but also greatly expands the range of various substrates, including electrophilic substituents which might be problematic in metal‐catalyzed procedures because of the possibility of side reactions or catalyst deactivation. According to their mechanistic studies, the amide bond's steric and electronic activation results in a lower strength of the amide resonance, which in turn causes the increased reactivity of *N*‐acyl‐glutarimides **5** and also the reactivity of this process is primarily controlled by the thermodynamic collapse of the tetrahedral intermediate **7** and the weakening of the amide bond.^[^ ^36^ ^]^

**Scheme 3:**
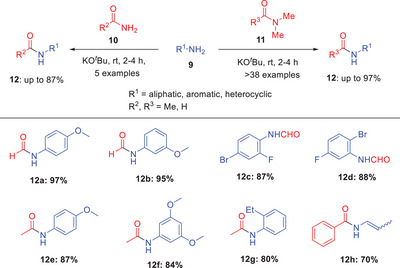
Potassium *tert*‐butoxide promoted transamidation of amides **10** and **11**.

In 2019, Dash and coworkers explored the potassium *tert*‐butoxide‐mediated transamidation of both primary **10** and tertiary amides **11** (Scheme 3).^[^ ^37^ ^]^ Various readily available inorganic bases were tried throughout the optimization process including KOH, K₂CO₃, Cs₂CO₃, KO*^t^* Bu, DBU, and NaOH. Among them, KO*^t^* Bu proved to be the most successful producing the maximum reaction yield of the desired transamidation product **12**. The strategy was applicable to a variety of amines **9**, including aryl, heteroaryl, and aliphatic amines, and transamidation processes were effectively carried out utilizing amides such as *N,N*‐dimethylformamide (DMF) and *N,N*‐dimethylacetamide (DMA). It was observed that the aniline derivatives with electron‐withdrawing substituents **12c** and **12** **d** needed longer time for the reaction and resulted in lower yields than those with electron‐donating groups **12a** and **12** **b**. Notably, an unusual reaction when cyclopropyl amine was employed in transamidation with tertiary amides. The reaction opened the cyclopropyl ring, resulting in the synthesis of enamide **12h,** respectively.

**Scheme 4:**
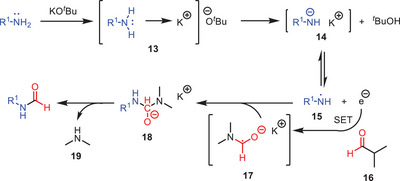
Plausible mechanism for the transamidation of amides **10** and **11** in the presence of KO*^t^* Bu.

Mechanistic investigations suggested that the reaction followed a free‐radical pathway, providing further insight into the reaction mechanism (Scheme 4).^[^ ^37^ ^]^ The reaction likely follows an electron‐transfer mechanism indicating a radical pathway for transamidation process. A Lewis basic amine coordinates with the potassium cation to form complex **13**, which quickly generates anion **14** through proton abstraction. Anion **14** undergoes single electron transfer (SET) to form amine radical **15**. This radical interacts with the amide **16** via another SET producing the radical anion intermediate **17**. Finally, the radical **15** couples with intermediate **17** to yield **18**. Finally, the intermediate **18** undergo an elimination of amino functionality to form the transamidation product **12**.

**Scheme 5:**
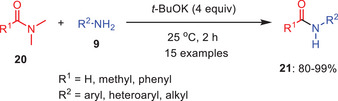
*^t^* BuOK initiated direct transamidation protocol of *N,N*‐dimethyl amides 20 with primary amines 9.

Similar to the recent protocol, A simple and successful approach for transamidating *N*,*N*‐disubstituted amides **20** with primary amines **9** was established, with potassium *tert*‐butoxide (*^t^* BuOK) serving as the catalyst (Scheme 5). In the case of *N*,*N*‐dimethylformamide (DMF), the transamidation process occurs efficiently at room temperature (25 °C) when *^t^* BuOK is employed as base in 4.0 equivalents, with DMF serving as the solvent under an inert environment, the process takes around 2 h to complete. Under optimal conditions, the transamidation reaction worked well with various amines. Both electron‐rich and electron‐deficient anilines gave high yields (90%–98%). Halogen‐substituted anilines (*ortho*/*para*) also performed well, except for *para*‐trifluoromethyl. Aliphatic and heterocyclic amines remained unchanged, while secondary amines like *N*‐methylaniline were less efficient due to potential side reactions. Notably, *α*‐naphthylaniline and *p*‐cyanoaniline also reacted smoothly.^[^ ^38^ ^]^

**Scheme 6:**
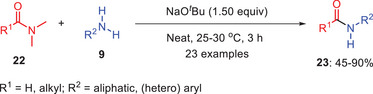
Sodium *tert*‐butoxide‐mediated transamidation of amides 22 with a range of primary amines 9 to amides 23.

In 2019, Zhang and coworkers successfully developed the transamidation of *N,N*‐dimethyl amides **22** with a range of primary amines **9** using sodium *tert*‐butoxide (NaO*^t^* Bu) as a base (Scheme 6).^[^ ^39^ ^]^ They tested a number of bases throughout the reaction optimization phase and when the reaction was carried out in an inert atmosphere, NaO*^t^* Bu produced the maximum conversion of amides to transamidation products **23**. It was observed that the course of reaction was influenced with stoichiometry of the base used. The versatility of the approach was highlighted by the high yields of amides that could be synthesized from aromatic, heteroaromatic, and aliphatic primary amines under optimal circumstances. Notwithstanding these benefits, there is a significant drawback to the methodology: substrates with aromatic substituents at the amide's α‐position are incompatible with NaO*^t^* Bu. Mechanistic investigations into the reaction showed that this transamidation does not proceed via a radical pathway, distinguishing it from other transamidation methods that employ bases like *^t^* BuOK, where radical mechanisms are typically involved.

**Scheme 7:**
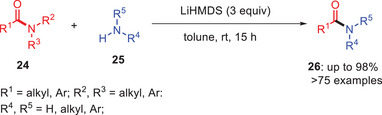
LiHMDS‐mediated transamidation of *N*‐Boc and *N*‐Ts amides **24** to amides **26**.

In 2018, Li et al. introduced a selective, metal‐free approach for transamidation of amides **26** with less‐nucleophilic amines **25** at room temperature using LiHMDS as a base and toluene as a solvent (Scheme 7). The method works well with both amides and esters as starting materials and accommodates a wide range of substrates including electron‐deficient and sterically hindered anilines along with functional groups like esters, halides, and heterocycle systems. It is also compatible with diverse amides such as *N,N*‐Boc_₂_ amides, *N*‐Ts, *N*‐Ms amides, and *N*‐acyl‐pyrroles.^[^ ^18^ ^]^

In 2019, the same research group extended their protocol to a broader range of substrates including amides derived from anilines substituted with halo, alkoxy, carboxyl, and alkyl groups. Notably, the reaction demonstrated compatibility with aromatic, aliphatic, and cyclic amines and consistently producing the desired transamide products **26** in excellent yields. This method was particularly remarkable for achieving the high chemo‐selectivity and its applicability to a wide range of *N,N*‐substituted amides regardless the type of substitution including pyrrolidinyl, morpholinyl, piperidinyl, and various *N,N*‐dialkyl amides. Additionally, the researchers investigated the transamidation of a model twisted bridged lactam (*τ* = 30.7°, χN = 49.7°, RE = 8.7 kcal/mol) and observed a strong preference for selective acyl N─C(O)N─C(O) bond cleavage over σ∖sigma C─NC─N bond scission.^[^ ^15^ ^]^

**Scheme 8:**
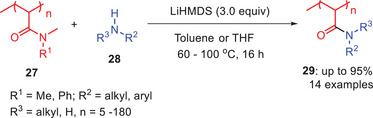
LiHMDS‐mediated direct transamidation of poly(*N*,*N*‐dimethylacrylamide) **27**.

Recently, Sumerlin and coworkers developed another base‐mediated transamidation of unactivated tertiary amides **27** to diversify poly (*N,N*‐dimethylacrylamide) (PDMA) and related acrylamide polymers **29** using three equivalents of LiHMDS (Scheme 8).^[^ ^40^ ^]^ This approach enhances atom economy, overcomes hydrolysis susceptibility and eliminates the requirement for extra functional monomer synthesis. Tolerance for functionalized amines, quantitative to moderate conversions and wide substrate compatibility were all observed. The chemo‐selective transamidation of copolymers with different amide reactivities including ultra‐high‐molecular‐weight copolymers were made possible in UHMW polymer synthesis. By demonstrating the possibility of small molecule chemistry in macromolecular modifications, this work broadens the tools available for precision polymer synthesis.^[^ ^40^ ^]^

**Scheme 9:**
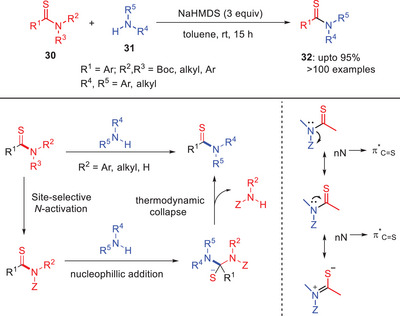
Base‐mediated transamidation of thioamides **30** using NaHMDS as a base.

Szostak and others developed a highly chemo‐selective approach for the transamidation of thioamides **30** via N─C(S) transacylation by employing weak nucleophilic anilines (Scheme 9).^[^ ^41^ ^]^ This approach allows site‐selective *N*‐Boc activation of primary and secondary thioamides by destabilizing the thioamide bond and enabling cleavage of the C(S)─N bond. The method works well with *N*‐thioacyl‐azoles and *N*‐Ar tertiary thioamides as selective* N*‐thioacyl transfer reagents. According to the DFT studies, thioamides highlight as “single‐atom substitution” isosteres of amides, with enhanced nN→π^∗^_C = S_ resonance due to increased polar form contributions. This resonance previously hindered direct transamidation. This approach effectively enables late‐stage derivatization of pharmaceuticals such as *N*‐mono‐Boc‐activated thioamides of probenecid, bexarotene, and ataluren reacted with functionalized anilines to yield transamidation products in good yields.^[^ ^41^ ^]^

**Scheme 10:**
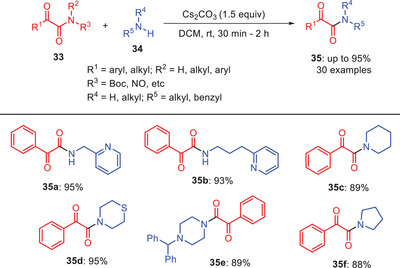
Base‐mediated transamidation of *α*‐ketoamides **33** with alkylamines **34** using Cs_2_CO_3_ as a base.

In 2021, Kandasmy and coworkers developed base‐mediated approach for the transamidation of *N*‐Boc α‐ketoamides **33** with various alkyl amines **34** in the presence of relatively weak base Cs₂CO₃ (Scheme 10).^[^ ^42^ ^]^ The reactions proceeded at room temperature and reaction products were obtained in good to excellent yields under optimized conditions. The methodology displayed broad substrate scope, functional group tolerance, and rapid conversion rates. Heteroaromatic amines such as 2‐aminomethyl pyridine and 2‐aminoethyl pyridine acted as effective nucleophiles by yielding products **35a** and **35b** with 95% and 93% yields, respectively. Similarly, acyclic and cyclic secondary amines including diethylamine, pyrrolidine, piperidine, morpholine, and *N*‐benzhydryl piperazine participated efficiently producing desired products **35c–35f** in high yields. However, no reaction occurred with less nucleophilic arylamines like aniline and its derivatives highlighting the critical role of amine nucleophilicity in the transamidation process.^[^ ^42^ ^]^

### 2.2 Acid‐Mediated Transamidation Reactions

**Scheme 11:**
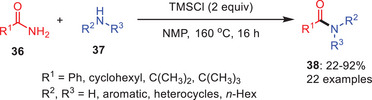
Trimethylsilyl chloride‐assisted transamidation of primary amides **36** to amides **38** using amines **37**.

There are a few acid‐mediated transamidation reactions that have been developed using by Lewis acid species. In 2019, Lee and coworkers demonstrated a Lewis acid‐mediated transamidation reaction of primary amides **36** was developed using trimethylsilyl chloride (TMSCl) as an activator (Scheme 11).^[^ ^43^ ^]^ This corresponding activation likely involves the conversion of the amide **36** into a more reactive intermediate, facilitating the nucleophilic attack by an amine **37**. This makes the primary amides more susceptible to undergo nucleophilic substitution. The reactions with primary amines produced the corresponding secondary amides **38** in moderate to excellent yields, when the transamidation was carried out in methylpyrrolidone (NMP) at 160 °C. The selection of solvent was found to play a crucial role in reactions involving secondary amines. Interestingly, the reaction facilitating the transamidation with secondary amines only in a mixture of NMP and chloroform, which is likely used to modulate the polarity and solvent effects.

**Scheme 12:**
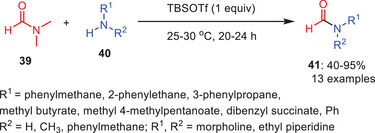
Lewis acid‐mediated transamidation of *N,N*‐dimethyl formamide **39** to amides **41**.

Sakurai et al. utilized *tert*‐butyldimethylsilyl triflate as an activator for developing an effective synthetic tool for the formylation of amines **40** with DMF **39** (Scheme 12).^[^ ^44^ ^]^ According to their optimization research, in case of activators, silane‐based Lewis acid activators such as TMSCl, TMSOTf, and TBSOTf excelled well compared with other Lewis acids including BF_3_, OEt_2_, AlCl_3_, TiCl_4_, and SnCl_4_. The amide products **41** exhibited a high tolerance toward aliphatic, aromatic, and heteroaromatic hydrazides along with the moderate‐to‐excellent yields. Furthermore, amines including heterocyclic, aromatic, and aliphatic amines performed well under the same circumstances. Nonetheless, the yield statistics showed that the yield of secondary amines was greater than that of primary amines. Importantly, imidazole and the supplementary equivalent of TBSOTf were also utilized in the reaction with amines/hydrazines that have a hydroxy group substituent.

### 2.3 Salt‐Mediated Transamidation Reaction

**Scheme 13:**
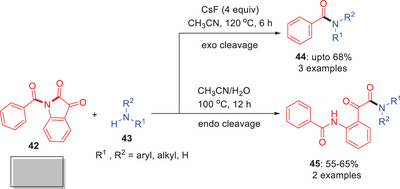
Selective *exo*/*endo* N─C bond cleavage in *N*‐acyl isatins **42**.

In 2019, Zeng and others demonstrated a divergent transamidation of *N*‐acylisatins **42** with secondary amines **43** using cesium fluoride as an activator (Scheme 13).^[^ ^45^ ^]^ *N*‐Acylisatins are reactive twisted amides (*τ* = 22.0°, χN = 13.9°) which generated unique products under varies conditions. Using four equivalents of CsF in acetonitrile at 120 °C provides *exo*‐transamidation products via formation of acyl fluoride intermediates. Alternatively, typical circumstances acetonitrile–water at 100 °C with no additives resulted in *endo* N─C(O) bond cleavage to generate distinct compounds **44** and **45**. Typically, the reaction between *N*‐acyl isatins and amines results in predictable transamination products through inner C─N bond cleavage. However, adding CsF unexpectedly promotes outer‐ring transamination favoring the cleavage of the outer C─N bond. This study introduces a novel strategy for achieving diverse chemical transformations from a single amide by selectively cleaving different C─N bonds.

### 2.4 Radical‐Initiated Transamidation Reactions

**Scheme 14:**
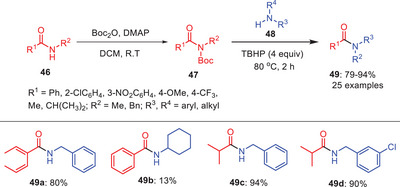
Transamidation of secondary amides **46** via C─N bond activation initiated by TBHP.

*tert*‐Butyl hydroperoxide (TBHP), a green and ecologically‐friendly radical initiator was used by Mishra et al. to achieve the transamidation of *N*‐Boc activated secondary amides (Scheme 14).^[^ ^46^ ^]^ In order to increase the electrophilicity amide's nitrogen and get it ready for later transamidation, this activation step was required. In the subsequent phase, TBHP facilitates the transamidation of the activated amide **47** with diverse amines **48**. According to their studies based on various amines, reaction occurs more readily with less hindered amine **49a** and **49** **b** than with α‐disubstituted hindered amine **49c** and **49** **d**. It is also observed that TBHP undergoes cross‐coupling with a broad range of substrates, a significant improvement over the limitations observed in the Et₃N‐catalyzed transamidation reactions as well as with nickel catalysis.

**Scheme 15:**
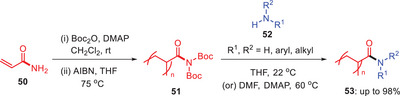
Metal‐free transamidation of *N*‐Boc‐activated polyacryl amides **51**.

In 2018, Hillmyer and colleagues developed a metal‐free transamidation process for poly‐*N,N*‐Boc₂‐acrylamides [poly(DBAm)] **51**, which enabled rapid synthesis of poly(acrylamides) **53** (Scheme 15).^[^ ^47^ ^]^ The procedure started with site‐selective Boc protection of the primary amide **50**, followed by radical polymerization with AIBN at 75 °C. Poly(DBAm) **51** was then treated with different amines **52** and catalytic DMAP in THF or DMF in order to generate poly(acrylamides) **53** in good to excellent yields. This approach has extensive substrate tolerance, including primary, α‐branched, sterically‐inhibited secondary amines and anilines. Its importance has been demonstrated in the synthesis of block copolymers and on‐demand gelation methods, indicating its ability to create a wide range of polymer structures. This method marks a substantial progress in polymer functionalization and material development.^[^ ^47^, ^48^ ^]^

### 2.5 Persulfate‐Mediated Transamidation Reaction

**Scheme 16:**
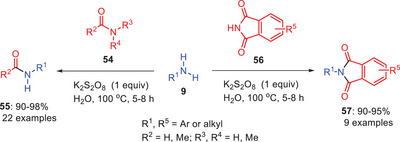
A metal‐free transamidation approach for amide–amine bond formation in aqueous medium.

Sawant and coworkers developed a green and efficient protocol for the transamidation of amides **54** and **56** with amines **9** using potassium persulfate in water (Scheme 16).^[^ ^49^ ^]^ The reaction is compatible with both conventional thermal heating and microwave irradiation allowing for greater flexibility in implementation. This protocol enables the preparation of a wide range of amides, demonstrating excellent substrate scope and selectivity and offering excellent yields. Interestingly, using this protocol l‐phenylalanine methyl ester hydrochloride was trans amidated and generating the *N*‐formyl product with no change in configuration or optical purity. This indicates the method's potential in asymmetric synthesis. Similarly, many other amino acids can also be transamidated using this protocol and same reaction and conditions.^[^ ^49^ ^]^

**Scheme 17:**
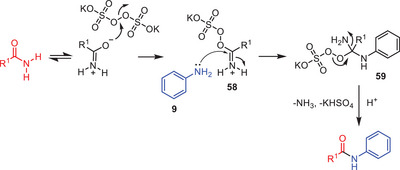
Mechanistic perspective of K₂S₂O₈ mediated transamidation.

From a mechanistic perspective, a time‐monitored experiment revealed the formation of various intermediates alongside the product within 30 min under conventional heating. Initially, the peroxy bond of K₂S₂O₈ cleaves upon mixing with an amide, forming intermediate **58** (Scheme 17). Subsequent addition of aniline **9** converts intermediate **58** to intermediate **59**, which ultimately releases ammonia and yields KHSO₄, leading to the formation of the transamidated product.

**Scheme 18:**
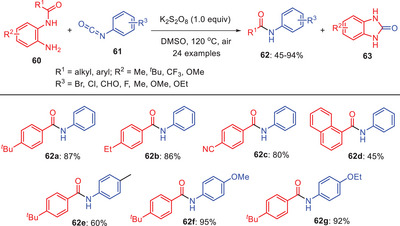
Mechano‐synthesis of secondary amides **62** from *N*‐(2‐aminophenyl) benzamides **60** via transamidation process.

Govindan et al. developed an effective synthetic approach for producing secondary amides **62** utilizing readily available *N*‐(2‐aminophenyl)benzamide **60** and phenyl isocyanate **61** in the presence of an additive potassium persulfate (Scheme 18). This approach features atom economy, practicality, and a one‐pot, two‐step reaction involving sequential nucleophilic/intramolecular addition and transamidation. Notably, the leaving group is recovered as a carbonylated *N*‐heterocycle, 1,3‐dihydrobenzimidazole‐2‐one **63**, which has pharmaceutical significance. Substrates with electron‐donating *para*‐substituents on the phenyl ring of *N*‐(2‐aminophenyl)benzamide yielded products **62a** and **62b** in 87% and 86% yields, respectively. Reactions with substituted *N*‐(2‐aminophenyl)benzamides and phenyl isocyanates produced double‐substituted amides **62c**–**f** with yields of 45%–95%. However, no product was obtained with aliphatic isocyanates. The by‐product, 1,3‐dihydrobenzimidazole‐2‐one **63** was recoverable under refined conditions and showed potential as a hypoglycemic agent and a versatile pharmaceutical structural motif.^[^ ^50^ ^]^

### 2.6 Iodide‐Mediated Transamidation Reaction

**Scheme 19:**
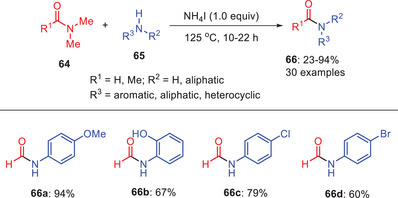
*N*‐acetylation of aromatic amines **65** by NH_4_I in the presence of DMA and DMF amides **64**.

Xie and coworkers developed a straightforward and efficient protocol for synthesizing amides **66** through the transamidation of amides **64** using amines **65** as nucleophile and NH₄I acting as a promoter (Scheme 19).^[^ ^51^ ^]^ Their procedure demonstrates a remarkable versatility in accommodating a wide range of aromatic, aliphatic, and heterocyclic amine substrates **65** which makes this approach highly adaptable for diverse synthetic needs. Notably, the reaction shows enhanced efficiency with anilines bearing electron‐donating substituents. These groups increase the nucleophilicity of the amine, facilitating smoother transamidation reactions and leading to higher yields of the desired amides **66a** and **66b**. However, when anilines contain electron‐withdrawing or deactivating groups, such as nitro (─NO₂) or halides (─Cl, ─Br), the nucleophilicity of the amine is significantly reduced. In the case of substrates like 4‐nitroaniline and 4‐methylaniline, the reaction went unsuccessful. These compounds are hindered either by steric factors or electronic effects, which compromise their ability to participate in the transamidation reaction efficiently. Furthermore, it was discovered that the presence of 2.0 equivalents of NH₄I was crucial for successful *N*‐acetylation when dimethylacetamide is employed as the acylating agent. Moreover, a noteworthy 29% yield of the antipyretic medication paracetamol was produced.^[^ ^51^ ^]^

### 2.7 Hypervalent Iodine‐Mediated Transamidation Reaction

**Scheme 20:**
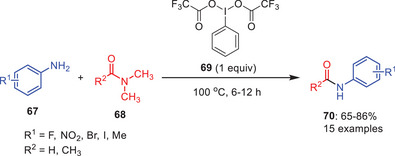
Hypervalent iodine‐mediated transamidation of carboxamides **68** with amines **67** using PIFA as an electrophile.

Hypervalent iodine reagents have shown some promising results in transamidation reactions.^[^ ^52^ ^]^ In 2022, Dandela and coworkers have developed hypervalent iodine mediated transamidation of amides in good yields using phenyliodine(III) bis(trifluoroacetate) (PIFA) **69** as an activator (Scheme 20).^[^ ^53^ ^]^ This novel approach takes advantage of PIFA's distinct features to enable the transamidation of dimethyl amide **68** with different amines **67**. One equivalent of PIFA was found sufficient to drive the reaction efficiently, making it a cost‐effective and practical alternative for amide synthesis. Under these conditions, an extensive range of aromatic amines, particularly those with fluoro substituents, reacted easily and afforded amide derivatives in excellent yields.

**Scheme 21:**
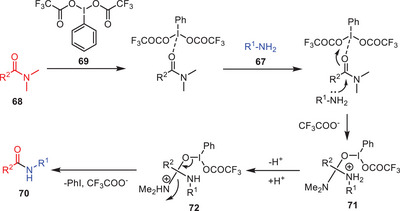
A plausible mechanism for PIFA‐induced transamidation of amides **68** with amine **67**.

A probable mechanism for PIFA‐promoted transamidation has been proposed. Initially, formamide **68** coordinates with PIFA **69**, facilitating a nucleophilic attack by amines **67** to form intermediate **71**. Proton transfer then generates intermediate **72**, followed by deamination to yield the desired amide product **70** (Scheme 21).^[^ ^53^ ^]^

### 2.8 Nitrite‐Mediated Transamidation Reaction

**Scheme 22:**
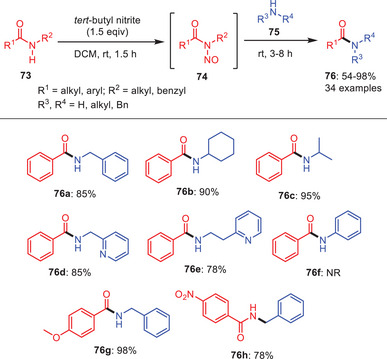
Transamidation of secondary amides **73** mediated by *tert*‐butyl nitrite.

In 2019, Kandasamy and coworkers developed an effective methodology for the transamidation reaction promoted by the use of *tert*‐butyl nitrite by reacting secondary amides **73** with a variety of amines **75** including primary, secondary, cyclic, and acyclic amines (Scheme 22).^[^ ^54^ ^]^ Where the secondary amide undergoes *N*‐nitrosation to form *N*‐nitrosamide intermediate **74** and it further undergoes nucleophilic addition with the respective amines **75**, followed by elimination offered transamidated products **76** in good‐to‐excellent yields. *N*‐Methyl benzamides showed good involvement in the reaction with amines **75a–c**. Interestingly, heteroaromatic amines like 2‐aminomethyl pyridine and 2‐aminoethyl pyridine proved to be effective nucleophiles in the transamidation process, yielding the desired products efficiently **76d** and **76** **e**. However, nucleophilic amines like aniline could not work well under given reaction conditions. In case of *N*‐alkyl benzamides, reactions worked well regardless of the substituents on the aryl ring and desired products were obtained with good to excellent yields **76g** and **76** **h**.

**Scheme 23:**
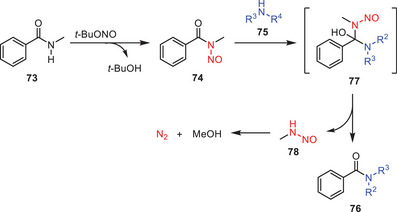
Plausible reaction mechanism for the *tert*‐butyl nitrite mediated transamidation of amides **73** to **76**.

Possible mechanism of the above transamidation reaction is shown in (Scheme 23). According to this, the reaction begins with the *N*‐nitrosation of the secondary amide **73** in the presence of *tert*‐butyl nitrite resulting in the formation of *N*‐nitrosamide **74**. This intermediate subsequently interacts with an amine **75** via nucleophilic addition to yield transitory intermediate **77**. Because of its instability, intermediate **77** undergoes an elimination process resulting in the desired transamidation product **76** along with the byproduct, alkyl *N*‐nitrosamine **78**.^[^ ^54^ ^]^

**Scheme 24:**
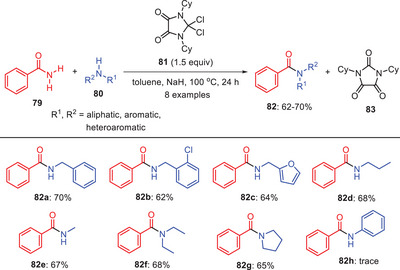
DCID‐mediated direct synthesis of amide **82** via transamidation of amides **79**.

Nasiri et al. reported an innovative and unconventional method for the transamidation of primary amides **79** using dichloroimidazolidinedione (DCID) **81** as a C─N bond activator (Scheme 24).^[^ ^55^ ^]^ This method stands out for its simplicity and efficiency as DCID alone mediates the reaction without the need for additional metal catalysts making it a cleaner and more environmentally friendly alternative. The study demonstrated a broad substrate scope and successfully synthesized secondary and tertiary amides **82** in good yields. Under optimized reaction conditions, the method exhibited a high degree of amine tolerance allowing smooth reactions with a wide variety of amines including benzylic, aliphatic, heteroaromatic, and secondary amines. However, the reaction showed limitations with less nucleophilic aniline by producing only trace amounts of the product **82** **h**. This limitation was attributed to the delocalization of the nitrogen lone pair within the aromatic ring, reducing its nucleophilicity and reactivity in the transamidation process. This approach highlights the potential of DCID as a versatile and effective mediator for transamidation.

**Scheme 25:**
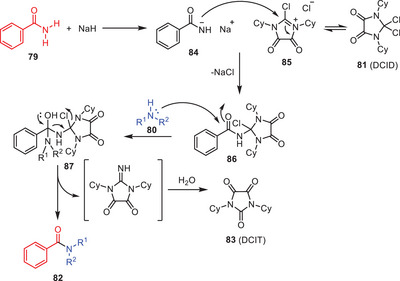
Reaction pathway for DCID mediated direct synthesis of amide **82** via transamidation protocol.

The proposed mechanism begins with the heterolytic cleavage of the C─Cl bond in DCID that facilitated by the electron donation from two nitrogen atoms adjacent to the quaternary carbon forming the 2‐chloro‐4,5‐dioxo‐imidazolinium chloride salt **85**. Next, a benzamide sodium salt acts as a nucleophile attacking the C2 position of intermediate **85** to produce intermediate **86**. This intermediate then undergoes a nucleophilic acyl substitution with amine functionality leading to the formation of a tetrahedral intermediate **87**. Finally, the elimination of 1,3‐dicyclohexylimidazolidine‐2,4,5‐trione (DCIT) **83** results in the formation of the desired transamidation products **82**. This stepwise process underscores the critical roles of nucleophilic attack and substitution in achieving the final product (Scheme 25).^[^ ^55^ ^]^

### 2.9 Microwave‐Assisted Transamidation Reaction

Microwave irradiation (MWI) is an unconventional, environmentally friendly energy source that has enhanced organic synthesis by enabling faster, more efficient, and sustainable transformations. It has been extremely useful in transamidation processes, in which one amide group is replaced by another. Recent advances include using potassium persulfate (K₂S₂O₈) for unactivated amides, heteropoly anion‐based ionic liquids for improved selectivity, ammonium salts for tertiary amides, and a catalytic system of iodine and hydroxylamine hydrochloride for primary, secondary, and tertiary amides. These achievements emphasize MWI's significance in advancing efficient, selective, and environmentally friendly techniques in contemporary chemistry.

**Scheme 26:**
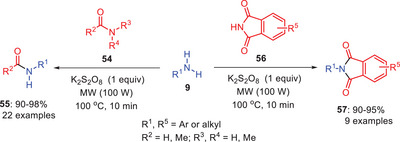
Microwave‐assisted synthesis of amides mediated by K_2_S_2_O_8_.

In 2015, Srinivas et al. developed a microwave‐assisted transamidation protocol for the synthesizing amides (Scheme 26). Utilizing various amines **9** and amides **54** and **56** mediated by K_2_S_2_O_8._ This method works well in both traditional heating (Scheme 16) and MW assisted synthesis. This peroxy reagent facilitates *N*‐protection of diverse amines, serving as a versatile protection strategy in synthetic organic chemistry. It offers an economical route to phthalimide‐protected derivatives, including amino acid derivatives, and is compatible with branched amines, nitrogen‐containing, and hydroxyl‐bearing substrates. To broaden the scope of this reaction, its application was explored for synthesizing various drugs and natural products such as phenacetin, paracetamol, and lidocaine, as well as the natural product piperine. This method is also suitable for synthesizing other drug intermediates or natural products, demonstrating its versatility.^[^ ^50^ ^]^

**Scheme 27:**
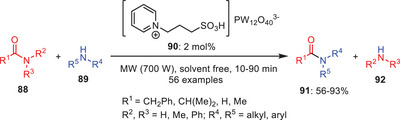
Ionic liquid‐mediated transamidation of carboxamides **88** with amines **89**.

Fu et al. introduced an innovative catalytic system for the transamidation of nonactivated carboxamides **88** with amines **89**, employing catalytic quantities of heteropolyacid‐based ionic liquids (HPAILs) **90** under microwave‐assisted and solvent‐free conditions (Scheme 27). This method displayed a broad substrate scope, successfully facilitating reactions with a diverse range of amines, including primary, secondary, and tertiary amines. The versatility of the approach was further highlighted by its compatibility with various amine types, demonstrating its potential for efficient and environmentally friendly synthesis of amides **91**.^[^ ^56^ ^]^

**Scheme 28:**
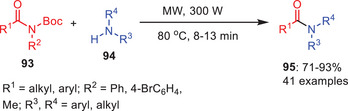
Chemoselective transamidation of secondary amides **93** with the utilization of microwave.

A microwave‐assisted, chemoselective approach has been established to transamidate Boc‐activated secondary carboxamides **93** with amines **94** in the absence of solvents, catalysts, and additives. Singh et al. (2023) used microwave irradiation to accomplish quick, efficient, and selective amide synthesis **95** (Scheme 28).^[^ ^57^ ^]^ They showed extensive application with a variety of *N*‐Boc‐activated aliphatic and aromatic amides as well as amines. When optimized, both weakly nucleophilic aromatic and aliphatic amines interacted smoothly, with yields dependent on amine structure and electrical characteristics. Electron‐donating groups on aryl amines produced high yields, but electron‐withdrawing groups produced moderate to good yields. Sterically hindered amines demonstrated slower reaction speeds and slightly decreased yields. According to mechanistic study, electron‐withdrawing amides are more reactive than electron‐donating amides.^[^ ^57^ ^]^

**Scheme 29:**
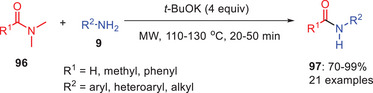
*^t^* BuOK initiated direct transamidation of *N*,*N*‐dimethyl amides **96** with primary amines **9** under microwave irradiation.

Tan et al. demonstrated the efficient protocol for the transamidation of *N*,*N*‐disubstituted amides **96** such as *N*,*N*‐dimethylacetamide (DMA), or dimethyl benzamide (DMB) with primary amines **9** was established, with *^t^* BuOK serving as the catalyst under microwave condition. Subsequently, the reaction also works with traditional method (Scheme 29). The reaction operates differently when DMA and DMB are employed as substrates, however. These changes need substantially higher temperatures, ranging from 110 °C–130 °C, and are carried out over 20–50 min utilizing microwave heating. The reaction also uses diglyme as a solvent in the presence of *^t^* BuOK to accomplish the required transamidation. During the substrate scope *ortho*‐substituted anilines are observed to provide a lower yield than all other anilines.^[^ ^38^ ^]^

## 3 Transamidation Reactions Involving Catalysis

### 3.1 Organocatalyzed Transamidation Reaction

**Scheme 30:**
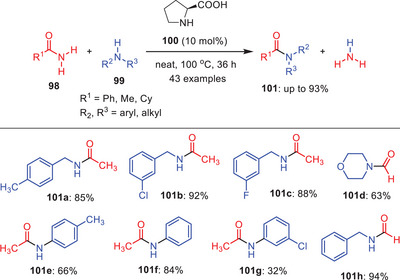
*l*‐roline‐mediated transamidation of carboxamides **98** with amines **99**.

Catalysis is one of the key tools of organic chemistry by which several organic transformations can be achieved with minimum negative on our environment.^[^ ^52^ ^]^ There are several catalytic approaches that have been developed to achieve the transamidation reaction in one decade. In this section, various transamidation reactions involving catalysis would be highlighted. In 2013, Adimurthy and coworkers developed a novel transamidation process catalyzed by *l*‐proline **100**, which allows for the selective and efficient transamidation of carboxamides **98** with various amine substrates **99** (Scheme 30).^[^ ^58a^ ^]^ This method was designed to target specific amide groups enabling their transformation with high precision and effectiveness. During the screening of substrate scope, the reaction demonstrated significant functional group compatibility when acetamide was used and it works well for corresponding amine derivates bearing electron rich and electron deficient substituents **101a‐c**. Additionally, the less nucleophilic anilines such as *p*‐methyl aniline **101e** and *m*‐chloroaniline **101** **g** provides better yields due to the delocalization of the nitrogen lone pair on aromatic ring system. Moreover, the formamide performs exceptionally well under these reaction conditions and produces transamidated products in good yields. This is attributed to the strong electrophilic nature of formamide, which enhances the reaction. However, this method proves ineffective for acyclic secondary amines like dibenzylamine and diisopropylamine. It was determined that the presence of free N─H and COOH groups in *l*‐proline is essential for the transamidation process to proceed efficiently.

**Scheme 31:**
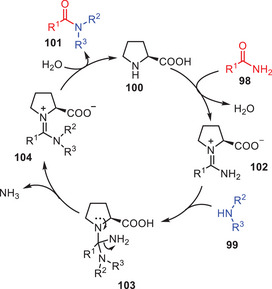
Plausible mechanism for *l*‐proline catalyzed transamidation of amides **98** with amines **99**.

Depending upon the literature reports^[^ ^58b^ ^]^ and experimental observations, the plausible reaction mechanism is given in (Scheme 31). According to that *l*‐proline **100** interacts with an amide **98** to make intermediate **102**, which then reacts with amine **99** to form tetrahedral intermediate **103**. The elimination of ammonia from **103** produces intermediate **104**, which when hydrolyze and generates the ultimate transamidation product **101** along with catalytic species. The process consists of three chemical steps‐ nucleophilic addition, elimination, and hydrolysis.

**Scheme 32:**
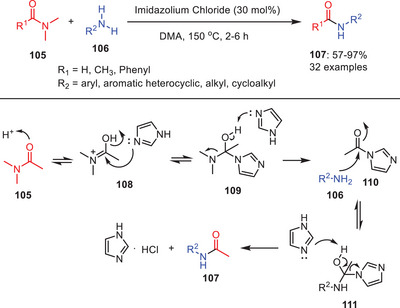
Imidazolium chloride catalyzed transamidation of tertiary amides **105** with amines **106**.

Another organocatalytic approach for transamidation of amides **105** has been developed by utilizing imidazolium chloride as a catalyst (Scheme 32).^[^ ^59^ ^]^ This approach has a wide range of flexibility in the terms of substituents in amides and amines and can potentially use with various primary amines **106** with tertiary amides **105** because it is compatible with an extensive spectrum of substrates. The aromatic primary amines bearing electron‐donating and withdrawing groups were tolerated well under the reaction conditions with efficient acetyl donor DMA. Whereas amines with electron‐donating groups shows better activity than withdrawing groups and anilines with nitro substituents shows less activity. It was noteworthy that the electronic properties of the substituent groups on the phenyl ring played an important role in the reaction.

**Scheme 33:**
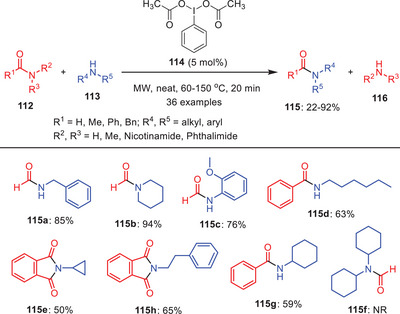
Hypervalent iodine‐catalyzed transamidation of carboxamides **112** with various amines **113**.

Hypervalent iodine catalysis has played a vital role in the progress of organic synthesis in the past two decades.^[^ ^60^ ^]^ In 2013, Singh and coworkers developed an effective route for the transamidation of carboxamides **112** with amines **113** using phenyliodine(III) diacetate (PIDA) **114** as catalyst under the microwave irradiations (Scheme 33).^[^ ^61^ ^]^ Substrates scope study suggests that the amide reactivity is affected by both steric and electronic functionalities. The higher electron‐donating capacity of alkyl groups over aryl groups accounts for the typical higher reactivity of aliphatic amides **115a‐c** over aromatic amides **115d–g**. Aliphatic amines are frequently more reactive than aromatic amines but both types of amines were employed successfully. The amide nitrogen atom's steric barrier might decrease reactivity. Steric hindrance causes aromatic amines with *ortho* substituents to have reduced yields **115c** although cyclohexylamine is entirely unreactive because of its large cyclohexyl group **115f**. Many different functional groups and substituents, such as Me, OMe, NO_2_, Cl, OH, and Br were tolerated in this catalytic protocol.

**Scheme 34:**
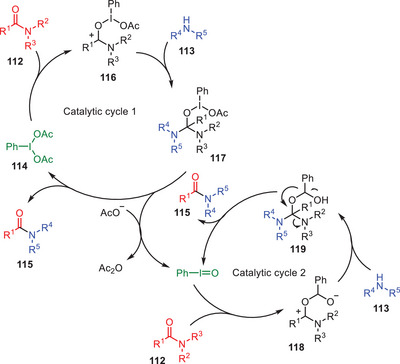
Catalytic cycle for the hypervalent iodine catalyzed transamidation of carboxamides **112** with various amines **113**.

The catalytic cycle was initiated with the activation of carbonyl functionality of an amide **112** by PIDA **114** to form the intermediate **116**, which reacts with amine **113** to form another intermediate **117**. Intermediate **117** undergoes the cleavage to yield transamidation product **115** along with formation of PhIO. PhIO further reacts with another molecule of an amide **112** to form the species **118** which undergo the nucleophilic addition of amine **113** to give the tetrahedral intermediate **119**. Finally, tetrahedral intermediate **119** undergoes the elimination step to yield transamidation product **115** with regeneration of catalytic species PhIO to continue the catalytic cycle (Scheme 34).^[^ ^61^ ^]^ Moreover, Hofmann rearrangement product was also observed in traces during this catalytic approach.

**Scheme 35:**
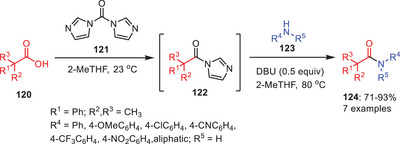
DBU‐catalyzed amidation of acyl imidazoles **122** to the amides **124**.

In 2009, an interesting approach for the transamidation of amides was developed by Vaidyanathan and coworkers using DBU as catalyst.^[^ ^62^ ^]^ In this approach, the starting acyl imidazoles **122** were synthesized in situ from corresponding acids which were transamidated with various secondary amines **123** in high yields (Scheme 35). Surprisingly, it significantly enhances reaction rates comparable to well‐established other catalysts. It was observed that the DBU catalysis was more effective for the anilines bearing electron‐withdrawing functionalities compared to the electron‐donating groups in the anilines. More likely, the DBU acts as the nucleophilic catalyst during this transformation.

**Scheme 36:**
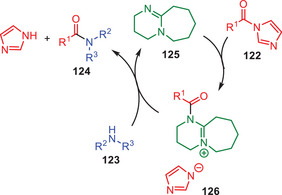
Catalytic cycle for DBU catalyzed transamidation of acyl imidazoles **122** to the amides **124**.

The catalytic cycle for DBU catalyzed transamination reaction of acyl imidazoles **122** to the amides **124** is depicted in (Scheme 36). The catalytic cycle was initiated by a nucleophilic attack of DBU **125** to the carbonyl functionality of amide **122** to form intermediate **126**. Intermediate **126** further reacts with the secondary amines **123** and gave the final products **124** along with regeneration of catalytic species to continue the catalytic cycle.

**Scheme 37:**
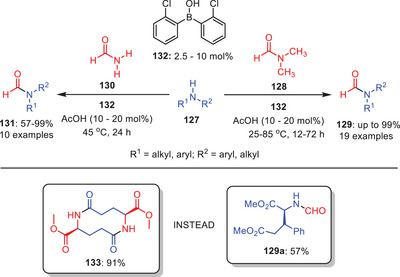
Borinic acid catalyzed transamidation of formamide **128** and **130** to amides **129** and **131**.

In 2016, a synthetic approach for the *N*‐formylation of amines **127** utilizing catalytic amounts of borinic acid **132** was investigated by Blanchet and coworkers (Scheme 37).^[^ ^63^ ^]^ Numerous primary and secondary amines as well as functionalized *α*‐amino esters were used to test the *N*‐formylation of amines. Based on their experimental study, they proposed that the reaction operates through cooperative catalysis, primarily driven by the increased Lewis acidity of the boron center rather than the enhanced Brønsted acidity of acetic acid. For chiral substrates, the observed racemization prompted a change in the formyl donor from dimethylformamide to formamide to minimize racemization and maintain it at a low level. Additionally, reaction scope was extended for the synthesis of isocyanides from primary amines. Interestingly, the methyl glutamate diester produced the macrolactam **133** in 91% yield rather than the predicted formamide **129a**. This data suggests an unexpected activation of the side chain, most likely involving the ester functional group. This activation might have enhanced intramolecular cyclization, favoring macrolactam synthesis over anticipated amidation. This unexpected result demonstrates the ester moiety's potential reactivity and significance in affecting the reaction route under the given circumstances.

**Scheme 38:**
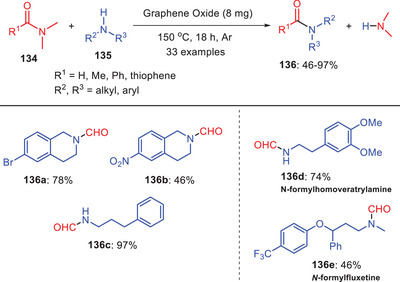
Graphene oxide‐catalyzed *N*‐formylation of amines **135** using transamidation process.

Ma et al. (2018) demonstrated the application of graphene oxide (GO) as a catalyst for amine *N*‐formylation. They used tertiary amides **134** and a variety of substituted secondary amines **135** to perform reactions in their investigation (Scheme 38).^[^ ^64^ ^]^ To avoid oxidation or other unintended reactions, the processes were carried out at 150 °C in an argon environment. This configuration demonstrated the efficiency of graphene oxide as a catalyst in enabling the transformation of the corresponding amide derivatives. However, the presence of an electron‐withdrawing group on the benzene ring of amine derivative negatively impacts this transformation and making it less effect In contrast, when other secondary amines whether cyclic or linear were employed as substrates, the reaction generally proceeded successfully. Using this strategy, fluoxetine, an antidepressant, was hydroformylated with a moderate yield of 46%. Unexpectedly, in primary amines, this reaction is highly dependent on the distance between the amino and phenyl groups. A remarkable 97% yield of **136c** is achieved when three methylene units separate these group. Due to the conjugation of nitrogen and benzene ring in the amines with hydroxyl substitution will decrease the nucleophilicity of nitrogen which makes the reaction unfavorable. They also concluded that the carboxyl groups on the graphene oxide surface are probably drive graphene oxide's catalytic activity, as opposed to the hydroxyl and epoxide groups.

**Scheme 39:**
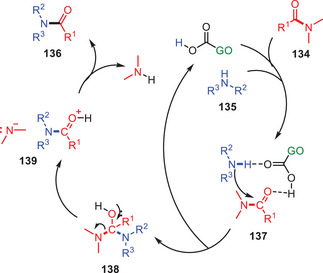
Proposed catalytic mechanism of GO catalyzed *N*‐formylation reaction of amines **135**.

Based on experimental findings and supporting literature, they suggest a viable mechanism for the *N*‐formylation process (Scheme 39). The carboxyl groups on the GO surface activate the amino and formyl groups resulting in the formation of an intermediate complex **137**. The carboxyl group's carbonyl moiety operates as Brønsted base and increasing the nucleophilicity of the amino group. The hydroxyl group interacts with the formyl group via hydrogen bonding and acts as a Lewis acid to activate the carbonyl group in DMF. The activated amino group attacks the activated carbonyl as nucleophile resulting in the formation of a tetrahedral intermediate **138**. Finally, the sterically congested intermediate **138** undergoes by proton transfer for releasing the target compound **136** and regenerating the active species.^[^ ^64^ ^]^

**Scheme 40:**
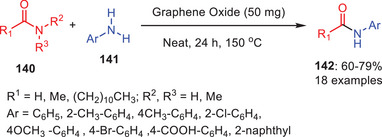
Graphene oxide‐mediated *N*‐formylation and *N*‐acetylation reactions.

In 2018, Bhattacharya et al. developed the novel synthetic route for the metal‐free transamidation using GO as a heterogeneous catalyst, they devised a method to change aliphatic amides **140** into aromatic amides **142** by reacting with various aromatic amines **141** (Scheme 40).^[^ ^65^ ^]^ For this transamidation process, graphene oxide function as an effective and recyclable catalyst because of its enormous surface area and many carboxylic acid groups on its edges which they stimulate the weakly electrophilic amide group, facilitating the following attack of the amine nucleophile via hydrogen bonding. Researchers used GO catalysts in reactions with both organic and inorganic solvents at different temperatures during process optimization, but the results fell short of what was anticipated. It was interesting to note that a high yield of the intended product was produced at 150 °C in solvent‐free circumstances with the same quantity of catalyst. By reacting aromatic anilines with various aliphatic carboxamides, this approach efficiently produced a range of formamide and acetamide compounds. However, the equivalent transamide product was not effectively produced by the reaction of carboxamides with an aliphatic amine.

**Scheme 41:**
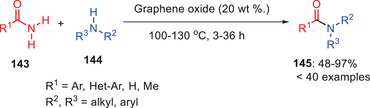
Graphene oxide‐assisted transamidation of unactivated amides **143**.

Patel et al. (2019) explored graphene oxide as a heterogeneous catalyst for transamidation reactions with systematically optimized the catalytic amounts, solvents and temperature (Scheme 41).^[^ ^66^ ^]^ Among tested carbon nanomaterials, graphene oxide (20%) at 130 °C yielded the best results and achieving 48%–97% efficiency with *aryl*‐substituted benzylamines and unsubstituted benzamides, though substituted benzamides showed lower yields. The method also worked well with branched‐chain, cyclic aliphatic amines, and formamides demonstrating compatibility with aryl, heteroaryl, and aliphatic substrates. The reaction tolerated phthalimides, thioamides, and selectively favored aliphatic amines, with urea yielding a dimeric transamide product. This approach highlighted graphene oxide's versatility and selectivity.

**Scheme 42:**
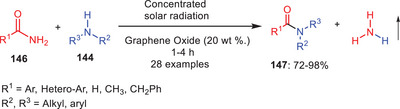
Graphene oxide catalyzed transamidation of amides **146** under solar radiation.

In 2020, the same research group invented a new pathway for the incorporation of solar energy in the amidation reactions thereby using concentrated solar radiation and graphene oxide as a catalyst. This synthetic process was established for the energy‐efficient and ecologically friendly transamidation of carboxamides and phthalimides **146** with different amines **144** (Scheme 42).^[^ ^67^ ^]^ This method not only minimizes energy consumption but also aligns with green chemistry principles by reducing the use of harmful reagents and promoting more sustainable chemical processes. Heat is produced when the molecules vibrate and spin due to the presence of infrared photons in solar radiation. The effective conversion of radiation into heat by concentrated sun energy allows for superheating at standard pressure. Solar radiation causes fast vibration and rotations which speeds up the rate of reaction by boosting molecular collisions and improving reactant‐reactant contact.

### 3.2 Inorganic Catalysis Transamidation Reaction

**Scheme 43:**
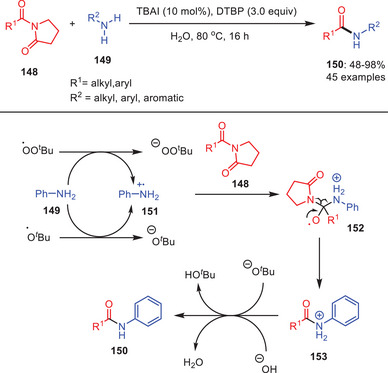
Aqueous promoted metal‐free transamidation of benzoylpyrrolidin‐2‐one **148** with amines **149**.

In recent years, inorganic catalysis has played a significant role in the development of organic synthesis.^[^ ^68^ ^]^ An aqueous approach of metal‐free transamidation was disclosed by Lee and co‐workers in which *tetra*‐*n*‐butylammonium iodide (TBAI) was used as catalyst and DTBP as radical initiator to achieve the reaction between various aromatic and aliphatic amines **149** with *N*‐acylpyrrolidin‐2‐one **148**. Reactions were working smoothly and transamidated products **150** were obtained in high yields (Scheme 43).^[^ ^69^ ^]^ The use of water as a solvent lowers the generation of hazardous waste and is a sustainable substitute for conventional organic solvents. Additionally, the reaction exhibits a broad substrate scope, and this flexibility allows for both aromatic and aliphatic amines to participate in the reaction to enable the formation of a diverse range of transamidated products. Interestingly, halide‐substituted aniline derivatives fared well in the process. Furthermore, in this typical reaction conditions, both benzoylpiperidin‐2‐one and benzoylazepan‐2‐one showed excellent reactivity. The *N*‐acyl lactam amides were utilized for the first time ever in the transamidation process so far. They also propose a radical reaction mechanism based on the control experiment data which includes the generation of intermediate acyl and aniline radicals during the process.

Based on their findings, they suggest the following chemical pathway: Di‐*tert*‐butyl peroxide (DTBP) interacts with TBAI to produce *tert*‐butoxyl radical and *tert*‐butoxide. *tert*‐Butoxide interacts with DTBP to produce *tert*‐butyl peroxy radical via iodine. Both radicals undergo single electron transfer with aniline resulting in aniline cation radical species **151**. This compound interacts with benzoylpyrrolidin‐2‐one **148** via a radical pathway to produce intermediate **152**, which then undergoes radical dissociation and releasing the pyrrolidin‐2‐one as a leaving group to yield the transamidated cation **153**. Finally, hydroxide or *tert*‐butoxide removes a proton from **153** resulting in the desired product **150**.^[^ ^69^ ^]^

**Scheme 44:**
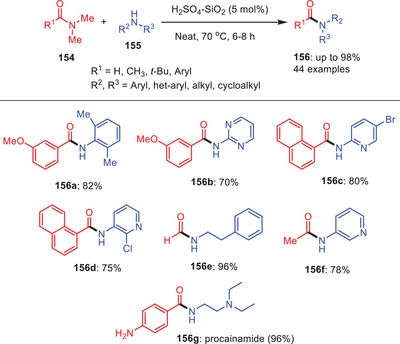
Acid‐catalyzed transamidation of amides **154** under solvent‐free conditions.

A solvent‐free methodology for the transamidation carboxamides **154** with amines **155** has been devised utilizing a catalytic amount of H_2_SO_4_‐SiO_2_ by Das and others (Scheme 44).^[^ ^70^ ^]^ This method works well with a wide range of aliphatic, aromatic, heteroaromatic and both cyclic and acyclic primary, or secondary amines. Based on the substrate scope, electron‐neutral, electron‐rich, electron‐deficient, aliphatic, and cyclic secondary amines produced the corresponding transamidation products **156** with high yields. Notably, sterically hindered arylamines interacted efficiently and produced transamidated product **156a**. Less reactive heteroaromatic compounds, such as 2‐aminopyrimidine **156b** formed the transamidation product in lower yields and with longer reaction times. It is worthnoting that amines with substitution such as bromo **156c** and chloro **156d** are well‐tolerated under these reaction conditions. Moreover, the catalytic reaction was expanded to include formylation and acetylation **156e** and **156** **f**. Additionally, this method paved way for the formation of commercially available drug procainamide **156g**.

**Scheme 45:**
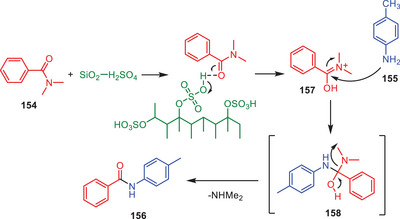
Plausible reaction mechanism for H_2_SO_4_‐SiO_2_‐mediated transamidation amides **154**.

Based on the experimental data and the known role of H_2_SO_4_/SiO_2_ as a proton‐transfer agent from its solid surface, a viable mechanism for the transamidation process has been postulated (Scheme 45). The process starts with the protonation and activation of the amide bond in *N,N*‐dimethyl benzamide **154** by the sulfonic acid group (─SO_3_H) on the surface of H_2_SO_4_/SiO_2_. This protonation produces a cationic intermediate **157** that is extremely reactive due to the partial positive charge on the carbonyl carbon. The cationic intermediate **157** is then attacked by an amine nucleophile **155**. This step includes the generation of a tetrahedral intermediate **158** in which the amine group binds to the carbonyl carbon. The intermediate then loses dimethylamine (NHMe_2_) as a leaving group forming the transamidation product **156**. The contribution of H_2_SO_4_/SiO_2_ in this process is critical since it not only aids in the initial protonation and activation of the amide bond but it also stabilizes the cationic intermediate enabling nucleophilic attack and eventual elimination. This solid acid catalyst serves as an efficient and reusable platform for the transamidation process, allowing for transformation under moderate circumstances.^[^ ^70^ ^]^

**Scheme 46:**
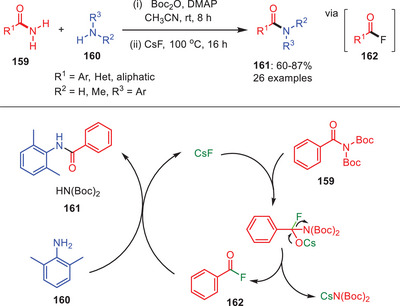
Transition‐metal‐free transamidation of primary amides **159** via tandem di‐Boc activation process.

In 2018, a significant one‐pot, transition‐metal‐free transamidation of primary amides **159** was developed by using cesium fluoride as catalyst by Zeng and others.^[^ ^71^ ^]^ This approach involves of two steps: selective *N*‐*tert*‐butoxycarbonylation (*N*‐Boc) of primary amides in order to generate reactive twisted *N,N*‐Boc₂‐amides, followed by CsF‐catalysed transamidation (Scheme 46). CsF facilitates the formation of an electrophilic acyl fluoride intermediate **162**, which undergoes nucleophilic attack by amines **160** to yield amides **161** along with the regeneration of CsF species to continue the catalytic cycle. This protocol operates under mild conditions, tolerates diverse functional groups, and accommodates sterically hindered substrates, making it efficient and practical. The process emphasizes the function of CsF in producing acyl fluoride and enhancing nucleophilic addition, resulting in excellent selectivity and reactivity without the need for transition metals.^[^ ^71^ ^]^

**Scheme 47:**
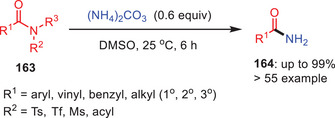
Synthesis of primary amides **164** by the transamidation of tertiary amides **163** using ammonium carbonate as catalyst.

Furthermore, Lee and coworkers invented an ammonia carbonate‐catalyzed catalytic approach for synthesis of primary amides **164** by the transamidation of tertiary amides **163** under moderate reaction conditions (Scheme 47).^[^ ^72^ ^]^ Utilizing (NH_4_)_2_CO_3_ in DMSO at 25 °C, tertiary amides containing *N*‐electron‐drawing groups such as sulfonyl, diacyl effectively generate primary amides with strong functional group tolerance. Furthermore, *N*‐tosylated lactams undergo ring opening to produce *N*‐tosylamido alkyl amides. This strategy is straightforward, adaptable, and works with a wide range of functional groups.

**Scheme 48:**
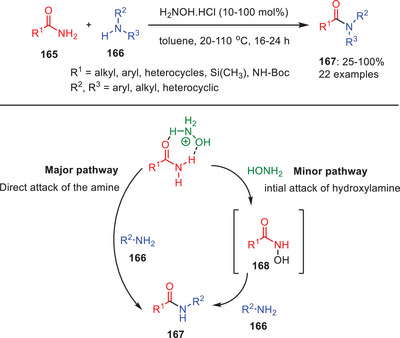
Hydroxylamine hydrochloride catalyzed transamidation of primary amides **165**.

In 2012, Allen and coworkers introduced an innovative method for the transamidation of primary carboxamides **165** with primary or secondary amines **166** to efficiently synthesize secondary or tertiary amides **167** (Scheme 48).^[^ ^73a^ ^]^ The key to this approach lies in the use of catalytic amounts of hydroxylamine hydrochloride which plays a crucial role in activating the typically inert and chemically stable primary amides. This activation allows the unreactive primary amide group to undergo nucleophilic substitution with the amine substrates leading to the formation of intermediate hydroxamic acid **168**, which further convert into secondary or tertiary amides. Subsequently, at lower temperatures, the quantity of catalyst required may increase. Among all the substituents, aliphatic amides gave the maximum conversions with less catalytic loading. Furthermore, the substrate screening revealed important insights such as the retention of Boc protecting groups throughout the process which permits specific acylation of a single nitrogen atom in mono‐protected diamines or selective coupling of an amine and an amino amide. Kinetic studies revealed a first‐order dependence on the concentration of hydroxylamine hydrochloride and the initial reaction rate. This indicates that one equivalent of hydroxylamine is involved in the rate‐determining step. As a result, the slow step of the mechanism could involve either the initial activation of the primary amide or the nucleophilic attack of the resulting complex.^[^ ^73b^ ^]^

**Scheme 49:**
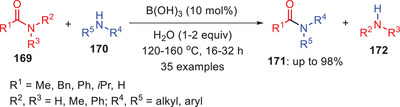
Boric acid catalyzed transamidation of carboxamides **169** with amines **170**.

Nguyen and coworkers developed a solvent‐free method for the transamidation of carbamides **169** with amines **170** using catalytic amounts of boric acid. Reactions were performed with 10 mol% of catalytic loading and reaction products were isolated in excellent yields (Scheme 49).^[^ ^74^ ^]^ This approach demonstrates broad applicability with various amines such as primary, secondary, tertiary, aliphatic, aromatic, cyclic, and acyclic and phthalimides. Its economic efficiency, simplicity, and functional group tolerance make it ideal for synthesizing valuable compounds like amides, lactams, peptides, ureas, and aza‐heterocycles, particularly for large‐scale applications. This method offers significant potential for advancing organic and pharmaceutical chemistry.

**Scheme 50:**
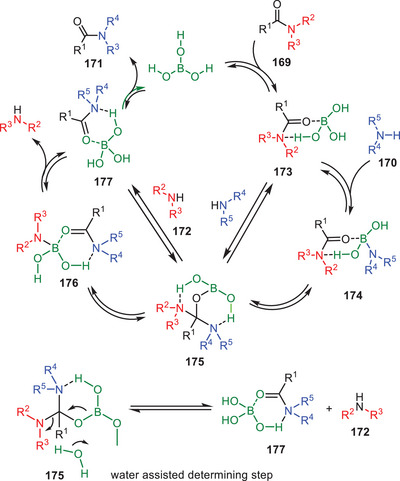
Proposed catalytic cycle for the boric acid catalyzed transamidation of amides **169**.

The experiment showed that boric acid catalyzes transamidation even for tertiary amides through a mechanism involving intermediate **173**. Boric acid acts as both a Lewis acid and coordinates with the oxygen of amide functionality and a hydrogen‐bond donor activating the amide for nucleophilic attack by amine **170**. The catalytic cycle begins with the formation of adduct **173** via proton transfer and B─O bond formation followed by amine attack to form intermediate **175**. The rate‐determining step is the conversion of aminal carbon to a boron‐bound amide facilitated by water which also prevents inactive boric acid aggregates. The cycle ends with the release of amide **171** (Scheme 50).^[^ ^74^ ^]^

**Scheme 51:**
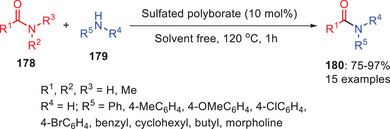
Sulfated polyborate catalyst transamidation of carboxamides **178** with amines **179**.

Chaturbhuj and coworkers describe catalytic study in which sulfated polyborate was used as a catalyst to achieve the transamidation of amides.^[^ ^75^ ^]^ Reaction was optimized, and 10 mol% of catalytic loading was found sufficient to catalyze the reaction and transamination products were isolated in high yields (Scheme 51). The reaction scope was studied with substrates having electron‐donating and withdrawing substituents on aniline, benzylamine, cyclohexylamine, and alicyclic secondary amines performed well with formamide proving slightly more effective than DMF. *N*‐acetylation using acetamide and DMA also succeeded with anilines, benzylic, and alicyclic amines, with acetamide yielding better results. The protocol was further extended to phthalimide transamidation with primary aromatic, benzylic, and alicyclic amines, producing *N*‐substituted phthalimides in high yields, offering a viable alternative for phthaloyl protection of primary amines.

**Scheme 52:**
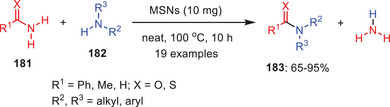
MSNs‐mediated transamidation of carboxamides and thiourea **181** under neat conditions.

Eidi et al. developed an efficient protocol for transamidation of amides **181** with amines **182** using mesoporous silica nanoparticles (MSNs) as a green, recyclable, and heterogeneous catalyst under solvent‐free conditions (Scheme 52).^[^ ^76^ ^]^ This method enables the synthesis of a variety of aromatic, aliphatic, and cyclic/acyclic amides **183** with yields ranging from 65% to 96%. Optimization studies showed that increasing the catalytic loading upto10 mol% provides the products in maximum yields while higher loading had no additional effect on the yield. The method demonstrated broad applicability, with successful transamidation of carboxamides and amines yielding aromatic, aliphatic, and heterocyclic amides efficiently. However, sterically hindered amines produced lower yields compared to aliphatic and benzylic amines. Additionally, the protocol extended to the transamidation of urea and thiourea with primary amines. This approach offers high yields, simplicity, short reaction times, and easy workups, presenting a practical and environmentally friendly alternative for other metal‐catalyst transamidation reactions.

## 4 Catalyst/Additive‐Free Transamidation Reaction

**Scheme 53:**
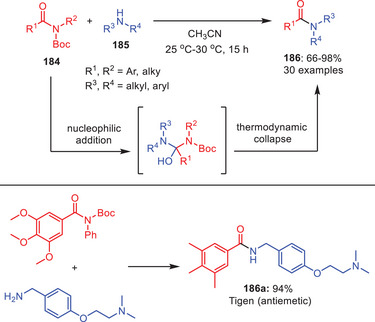
Transamidation of *N*‐Boc amides **184** without using any additive.

In 2019, Szostak and coworkers demonstrated a simple and highly chemo‐selective protocol for the transamidation of *N*‐*tert*‐butoxycarbonylated secondary amides **184** (Scheme 53).^[^ ^77^ ^]^ Herein, various substituted *N*‐Boc amides **184** were reacted with wide range of secondary amines **185** in acetonitrile. Remarkably, the circumstances under which this reaction takes place are quite moderate and there is no need for any additives. There is a possibility that the efficiency of the transamidation process might be improved by stabilizing the tetrahedral intermediate in a transition state that is more polar. The crucial issue here is balancing the enhanced rate of nucleophilic addition to the *N*‐Boc activated N─C(O) amide bond with the amide bond deprotection by *N*‐Boc cleavage, which deactivates the amide bond against mild nucleophilic addition. The effectiveness of this approach has been proved in the direct synthesis of marketed antiemetic drug, *Tigan* **186a**.

**Scheme 54:**
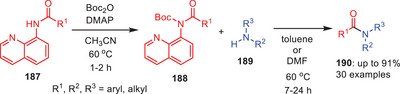
Transamidation of 8‐aminoquinoline amides **187** through intermediate *N*‐AcylBoc‐Carbamates **188**.

In 2018, Verho and coworkers developed a two‐step, metal‐free transamidation protocol of 8‐aminoquinoline amides **187**.^[^ ^78^ ^]^ After destabilizing the ground state with *N*‐Boc, the secondary amides **188** were treated with amines **189** in toluene or DMF at 60 °C and reaction products were isolated on average‐to‐good yields (Scheme 54). The approach works with sensitive functional groups, less nucleophilic amines, and a variety of alkylamines. By producing functionalized amide products in high to outstanding yields, this method greatly expands the use of 8‐aminoquinoline amides as C─H functionalization intermediates. This method offers a productive, metal‐free substitute for altering 8‐aminoquinoline amides.

**Scheme 55:**
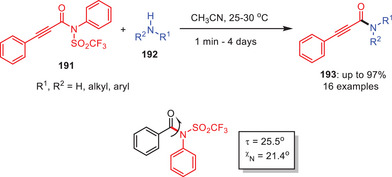
Metal‐free transamidation of *N*‐triflylpropynamides **191** via twisted *N*‐Tf‐amides.

In 2019, Fiore and Maas reported a metal‐free transamidation of *N*‐Tf‐propynamides **191** (Tf = triflyl) with amines **192** in good‐to‐excellent yields under moderate conditions (Scheme 55).^[^ ^79^ ^]^ Reaction showed good functional group tolerance with both aliphatic and aromatic amines. *N*‐Tf‐propynamides serve as *N*‐acylation precursors which can be conveniently synthesized from terminal alkynes, isocyanates, and triflic anhydride in a single step. Mechanistically, the process is most likely to involve acyl substitution, however ketene‐type intermediates cannot be eliminated. The triflimide group highly activates the amides, making *N*‐Tf‐propynamides much more reactive than *N*‐Ts amides.^[^ ^80^ ^]^ This study demonstrates the usefulness of triflyl activation for efficient, selective amide bond production.

**Scheme 56:**
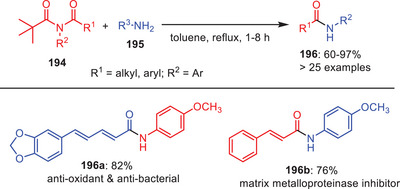
One‐pot transamidation of *N*‐pivaloyl activated amides **194**.

Recently, Rajan and Rajendran described a novel one‐pot transamidation process for the *N*‐pivaloyl‐activated amides **194** utilizing weak nucleophilic anilines **195** which requires no catalysts, bases, or additives. Transamidation products **196** were obtained in moderate‐to‐excellent yields by activating amides in situ with the pivaloyl group and reacting them with anilines at toluene reflux temperature (Scheme 56).^[^ ^81^ ^]^ In this approach, transamidation of various substrates was achieved effectively with different electrically varied aryl amines. Additionally, it paved way for the synthesis of various bioactive drugs **196a** and **196b** in good yields. Competitive studies demonstrated that planar *N*‐pivaloyl‐activated secondary amides and nonplanar *N*‐pivaloyl‐activated tertiary amides have comparable reactivity. The approach converts planar, resonance‐stabilized *N*‐activated secondary amides into stable precursors for transamidation, providing a flexible and chemo‐selective tool for chemical synthesis.

**Scheme 57:**
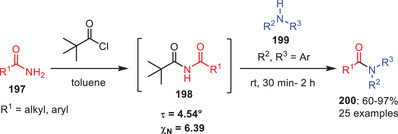
Catalyst and additive‐free transamidation of *N*‐pivaloyl activated amides **198**.

Later on, the same group achieved the transamidation of planar (twist angle *τ* = 4.54°) and resonance‐stabilized *N*‐pivaloyl‐activated amides **198** without using any catalyst, base or additives at room temperature with alkyl amines **199** in short reaction times (0.5–2 h) and yields of 60%–97% (Scheme 57).^[^ ^14^ ^]^ The amidic resonance evidenced by C─N (1.374 Å) and C═O (1.222 Å) bond lengths does not hinder reactivity and the method tolerates amines with protic groups like hydroxy and carboxylic acids. A one‐pot protocol enables the transamidation of these planar (*τ* = 4.54°, χN = 6.39) and resonance‐stabilized amides, accommodating a wide range of alkyl amines including those with protic functionalities. Competitive and control studies reveal that steric hindrance influences chemo‐ and regioselectivity, highlighting the feasibility of nucleophilic addition to such amides under mild and catalyst‐free conditions. This strategy was also applied for the synthesis of natural products and drug molecules. Moreover, it was extended to other nucleophiles offering an environmentally benign approach to functionalizing *N*‐pivaloyl‐activated amides.

## 5 Solvent‐Free Transamidation Reactions

**Scheme 58:**
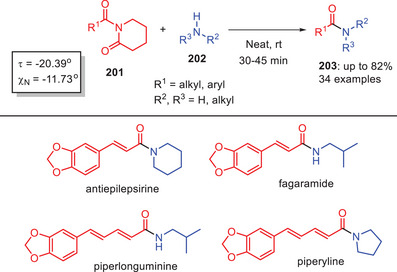
Transamidation of *N*‐acyl‐2‐piperidones **201** under solvent‐free condition.

In 2019, Subramani and Rajendran identified *N*‐acyl‐2‐piperidones **201** as highly effective substrates for metal‐free transamidation under moderate, solvent‐free conditions at room temperature (Scheme 58). The X‐ray structure of a vinyl derivative showed a considerably twisted amide bond (*τ* = 20.4°, χ_N_ = 11.7°) indicating structural characteristics that increase reactivity. This methodology displayed a broad substrate scope accommodating a variety of imide and amine substitutions including functionalized amines with carboxylic acid, ester, and hydroxyl groups functionalities that are frequently incompatible with metal‐catalyzed procedures. Importantly, the approach worked well with physiologically relevant substrates including amino acids and amino alcohols. The flexibility of *N*‐acyl‐2‐piperidones was further demonstrated by the synthesis of bioactive natural compounds such as antiepilepsirine, piperlonguminine, piperyline, and fagaramid*e*. This approach provides a versatile and environmentally benign alternative to amide bond production in organic synthesis.^[^ ^16^ ^]^

**Scheme 59:**
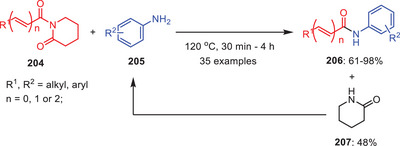
Transamidation protocol for weakly nucleophilic aromatic amines **205**.

In 2022, the same research group reported another catalyst, additive, base, and solvent‐free protocol for the transamidation of *N*‐acyl‐2‐piperidinones **204** with weakly nucleophilic aromatic amines **205** under melt conditions (Scheme 59).^[^ ^17^ ^]^ This environmentally benign methodology offers the transamidation products in good‐to‐excellent yields and demonstrates chemo‐selectivity, tolerating well the protic groups like carboxylic acids and hydroxy functionalities. The process is displayed for the synthesis of the bioactive natural product Avenanthramide‐A and highlights atom economy by isolating and reusing the by‐product, 2‐piperidinones for resynthesizing starting material. The protocol eliminates the need for protection‐deprotection steps offering a streamlined and sustainable approach to synthesizing amides, peptides, and amide‐based drugs. Its operational simplicity, broad applicability, and eco‐friendly nature make it a versatile tool for organic synthesis.

**Scheme 60:**
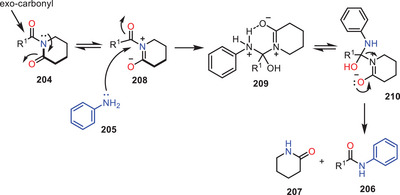
Plausible reaction mechanism for transamidation of *N*‐acyl‐2‐piperidinones **204** with aromatic amines **205**.

A proposed mechanism involves *N*‐acyl‐2‐piperidinones **204**, where amide bond twist aligns the imide's nitrogen lone pair and *exo*‐carbonyl in different planes, enhancing resonance with the ring carbonyl **208**. It increases *exo*‐carbonyl electrophilicity, enabling nucleophilic attack by aniline forming a C─N bond **209**. Proton exchange yields intermediate **210** followed by C─N (lactam) bond cleavage, producing the transamidation product **206** and 2‐piperidinone as a by‐product **207** (Scheme 60).^[^ ^17^ ^]^

**Scheme 61:**
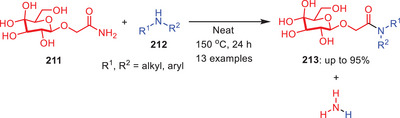
Catalyst‐free transamidation of unprotected glycosyl carboxamides **211** under neat reaction conditions.

A catalyst and solvent‐free transamidation of glycosyl carboxamides has been developed by Laclef and others resulting in a green and efficient way for synthesis glycosyl amides.^[^ ^82^ ^]^ The reaction tolerates a wide range of amines and unprotected carbohydrates producing the reaction products **213** with good‐to‐high yields while producing primarily ammonia as a byproduct (Scheme 61). Although epimerization at the α‐position of the amide group is limited but this hypothesized process includes intermolecular hydrogen bond activation. This approach shows potential for producing glycolipids, glycoconjugates, and bioactive molecules such the phosphoglycolipids GLP 1 and GLP 2. Furthermore, this methodology was applied to glycopeptide synthesis by incorporating glycosyl carboxamides with amino acids.^[^ ^82^ ^]^

**Scheme 62:**
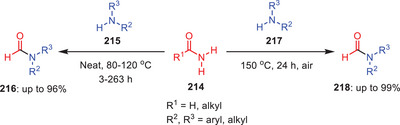
Transamidation of formamide **214** under neat reaction conditions.

In 2014, Legros and others introduced an approach for the transamidation that involved the reaction of formamide **214** with various amines **215** under heating condition without requiring any catalyst or solvent (Scheme 62).^[^ ^83^ ^]^ This approach offered an environmentally friendly and neutral pathway for the formylation of primary and secondary amines aligning with green chemistry principles. A notable feature of this method was its simplicity and efficiency, as the resulting amide product could be directly converted into monomethylamine through a one‐pot sequence further enhancing its utility in chemical synthesis.^[^ ^83^ ^]^ In 2018, a similar approach was reported to extend its application by Gong and coworkers.^[^ ^84^ ^]^ In this approach, authors demonstrated the transamidation of weak nucleophilic aromatic amines **217** with formamide derivatives **214** as well as the reaction of low‐reactive tertiary amides with aliphatic amines. These advancements highlighted the versatility and robustness of the catalyst‐ and solvent‐free transamidation process displaying its ability to accommodate a broader range of substrates and reaction conditions while maintaining efficiency and sustainability.^[^ ^84^ ^]^

**Scheme 63:**
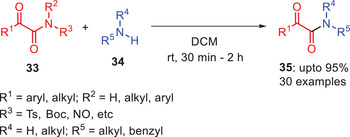
Transamidation of *α*‐ketoamides **33** with alkylamines **34** under neat reaction condition.

Singh et al. developed a catalyst, base, and additive‐free method for the transamidation of *N*‐tosyl α‐ketoamides **33** with alkyl amines **34** at room temperature (Scheme 63).^[^ ^42^ ^]^ Transamidation reactions were working smoothly and reaction products **35** were achieved in good‐to‐excellent yields. During these transformations, various functional groups were successfully tolerated. The protocol also worked in the presence of a base offering a simple, efficient, and environmentally friendly approach for synthesizing *α*‐ketoamide derivatives.^[^ ^42^ ^]^

## 6 Conclusion

In conclusion, both academic and industrial regions have expressed a strong interest in amide synthesis. Amides are fundamental building components for many proteins and useful materials. Transamidation reactions were explored in order to discover new synthetic procedures. This has made it feasible to convert readily available amide derivatives into a wide range of target amides. This technique offers a modern solution to the challenge of interconverting functional groups as well as a pathway for the successful synthesis of target molecules. Transamidation processes are especially intriguing and scalable strategies that provide new possibilities for chemical synthesis in a range of fields. Transamidation facilitates the formation of novel amide bonds.

## Author Contributions

Niharan Sivaraj was responsible for identifying and selecting relevant literature and drafting the manuscript. Fateh V Singh and Toshifumi Dohi contributed to the conceptualization, review, and final editing of the manuscript.

## Conflict of Interests

The authors declare no conflict of interest

## Data Availability

Data sharing is not applicable to this article as no new data were created or analyzed in this study.

## References

[1] A. Greenberg , C. M. Breneman , J. F. Liebman , The Amide Linkage: Structural Significance in Chemistry, Biochemistry, and Materials Science, Wiley InterScience, Chichester 2000.

[2] K. Tani , B. M. Stoltz , Nature 2006, 441, 731–734. 16760973 10.1038/nature04842

[3] V. R. Pattabiraman , J. W. Bode , Nature 2011, 480, 471–479. 22193101 10.1038/nature10702

[4] S. D. Roughley , A. M. Jordan , J. Med. Chem. 2011, 54, 3451–3479. 21504168 10.1021/jm200187y

[5] D. G. Brown , J. Bostrom , J. Med. Chem. 2016, 59, 4443–4458. 26571338 10.1021/acs.jmedchem.5b01409

[6] A. B. Hughes , Amino Acids, Peptides, and Proteins in Organic Chemistry, Building Blocks, Catalysis and Coupling Chemistry, Wiley‐VCH, Weinheim 2011, 3.

[7] K. Marchildon , Macromol. React. Eng. 2011, 5, 22–54.

[8] B. M. Trost , I. Fleming , Comprehensive Organic Synthesis: Selectivity, Strategy and Efficiency in Modern Organic Chemistry, Elsevier, Amsterdam 1992, 9.

[9] J. R. Dunetz , J. Magano , G. A. Weisenburger , Org. Process Res. Dev. 2016, 20, 140–177.

[10] P. Acosta‐Guzmán , A. Mateus‐Gómez , D. Gamba‐Sánchez , Molecules 2018, 23, 2382. 30231486 10.3390/molecules23092382 PMC6225162

[11] C. L. Allen , J. M. Williams , Chem. Soc. Rev. 2011, 40, 3405–3415. 21416075 10.1039/c0cs00196a

[12] R. M. De Figueiredo , J. S. Suppo , J. M. Campagne , Chem. Rev. 2016, 116, 12029–12122. 27673596 10.1021/acs.chemrev.6b00237

[13] W. D. Loomis , P. K. Stumpf , D. Stickstoffumsatz , Nitrogen Metabolism 1958, 262–276.

[14] I. A. P. S. Rajan , S. Rajendran , Org. Biomol. Chem. 2023, 21, 4760–4765. 37248856 10.1039/d3ob00418j

[15] G. Li , C. L. Ji , X. Hong , M. Szostak , J. Am. Chem. Soc. 2019, 141, 11161–11172. 31203613 10.1021/jacs.9b04136

[16] M. Subramani , S. K. Rajendran , Eur. J. Org. Chem. 2019, 22, 3677–3686.

[17] I. A. P. S. Rajan , M. Subramani , G. Pushparathinam , S. Rajendran , Asian J. Org. Chem. 2022, 11, 202200378.

[18] G. Li , M. Szostak , Nat. Commun. 2018, 9, 4165. 30302003 10.1038/s41467-018-06623-1 PMC6178361

[19] M. V. Lee , S. R. Raga , Y. Kato , M. R. Leyden , L. K. Ono , S. Wang , Y. Qi , J. Mater. Res. 2017, 32, 45–55.

[20] J. Cretenoud , S. Galland , C. J. Plummer , V. Michaud , A. Bayer , N. Lamberts , B. Hoffmann , H. Frauenrath , Appl. Polym. Sci. 2017, 134, 44349.

[21] R. Király , K. Thangaraju , Z. Nagy , R. Collighan , Z. Nemes , M. Griffin , L. Fésüs , J. Amino acids 2016, 48, 31–40. 10.1007/s00726-015-2063-5 26250429

[22] V. H. Pham , H. Maaroufi , C. Balg , S. P. Blais , N. Messier , P. H. Roy , F. Otis , N. Voyer , J. Lapointe , R. Chenevert , FEBS Lett. 2016, 590, 3335–3345. 27586694 10.1002/1873-3468.12380

[23] L. Pauling , The Nature of the Chemical Bond—An Introduction to Modern Structural Chemistry, Cornell University Press, New York 1960.

[24] K. Sakthivel , S. Niharan , F. V. Singh , J. Organomet. Chem. 2024, 1018, 123290.

[25] H. Sheng , R. Zeng , W. Wang , S. Luo , Y. Feng , J. Liu , W. Chen , M. Zhu , Q. Guo , Adv. Synth. Catal. 2017, 359, 302–313.

[26] a) S. E. Eldred , D. A. Stone , S. H. Gellman , S. S. Stahl , J. Am. Chem. Soc. 2003, 125, 3422–3423; 12643691 10.1021/ja028242h

[27] M. Shi , S. C. Cui , Synth. Commun. 2005, 35, 2847–2858.

[28] M. Tamura , T. Tonomura , K. I. Shimizu , A. Satsuma , Green Chem. 2012, 14, 717–724.

[29] a) N. A. Stephenson , J. Zhu , S. H. Gellman , S. S. Stahl , J. Am. Chem. Soc. 2009, 131, 10003–10008; 19621957 10.1021/ja8094262

[30] S. C. Ghosh , C. C. Li , H. C. Zeng , J. S. Ngiam , A. M. Seayad , A. Chen , Adv. Synth. Catal. 2014, 356, 475–484.

[31] a) E. L. Baker , M. M. Yamano , Y. Zhou , S. M. Anthony , N. K. Garg , Nat. Commun. 2016, 7, 11554; 27199089 10.1038/ncomms11554 PMC4876455

[32] Y. Shimizu , H. Morimoto , M. Zhang , T. Ohshima , Angew. Chem., Int. Ed. 2012, 51, 8564–8567. 10.1002/anie.201202354 22782540

[33] L. Becerra‐Figueroa , A. Ojeda‐Porras , D. Gamba‐Sanchez , J. Org. Chem. 2014, 79, 4544–4552. 24758779 10.1021/jo500562w

[34] T. Zhou , G. Li , S. P. Nolan , M. Szostak , Org. Lett. 2019, 21, 3304–3309. 30990697 10.1021/acs.orglett.9b01053

[35] Y. Liu , S. Shi , M. Achtenhagen , R. Liu , M. Szostak , Org. Lett. 2017, 19, 1614–1617. 28290204 10.1021/acs.orglett.7b00429

[36] Y. Liu , M. Achtenhagen , R. Liu , M. Szostak , Org. Biomol. Chem. 2018, 16, 1322–1329. 29393316 10.1039/c7ob02874a

[37] T. Ghosh , S. Jana , J. Dash , Org. Lett. 2019, 21, 6690–6694. 31429570 10.1021/acs.orglett.9b02306

[38] Z. Tan , Z. Li , Y. Ma , J. Qin , C. Yu , Eur. J. Org. Chem. 2019, 4538–4545.

[39] R. Zhang , J. C. Zhang , W. Y. Zhang , Y. Q. He , H. Cheng , C. Chen , Y. C. Gu , Synthesis 2020, 52, 3286–3294.

[40] L. Trachsel , D. Konar , J. D. Hillman , C. L. Davidson IV , B. S. Sumerlin , J. Am. Chem. Soc. 2024, 146, 1627–1634. 38189246 10.1021/jacs.3c12174

[41] G. Li , Y. Xing , H. Zhao , J. Zhang , X. Hong , M. Szostak , Angew. Chem., Int. Ed. 2022, 61, e202200144. 10.1002/anie.202200144 PMC8983593 35122374

[42] S. Singh , S. Popuri , Q. M. Junaid , S. Sabiah , J. Kandasamy , Org. Biomol. Chem. 2021, 19, 7134–7140. 34355726 10.1039/d1ob01021b

[43] S. Yu , K. H. Song , S. Lee , Asian J. Org. Chem. 2019, 8, 1613–1616.

[44] M. Sakurai , R. Kawakami , N. Kihara , Tetrahedron Lett. 2019, 60, 1291–1294.

[45] L. Xiong , R. Deng , T. Liu , Z. Luo , Z. Wang , X. F. Zhu , H. Wang , Z. Zeng , Adv. Synth. Catal. 2019, 361, 5383–5391.

[46] A. Mishra , S. Chauhan , P. Verma , S. Singh , V. Srivastava , Asian J. Org. Chem. 2019, 8, 853–857.

[47] M. B. Larsen , S. E. Herzog , H. C. Quilter , M. A. Hillmyer , ACS Macro Lett. 2018, 7, 122–126. 35610928 10.1021/acsmacrolett.7b00896

[48] M. B. Larsen , S. J. Wang , M. A. Hillmyer , J. Am. Chem. Soc. 2018, 140, 11911–11915. 30215257 10.1021/jacs.8b07542

[49] M. Srinivas , A. D. Hudwekar , V. Venkateswarlu , G. L. Reddy , K. A. Kumar , R. A. Vishwakarma , S. D. Sawant , Tetrahedron Lett. 2015, 56, 4775–4779.

[50] K. Govindan , N. Q. Chen , A. Jayaram , W. Y. Lin , New J. Chem. 2024, 48, 1103–1107.

[51] J. Chen , J. Jia , Z. Guo , J. Zhang , M. Xie , Tetrahedron Lett. 2019, 60, 1426–1429.

[52] F. V. Singh , S. E. Shetgaonkar , M. Krishnan , T. Wirth , Chem. Soc. Rev. 2022, 51, 8102–8139. 36063409 10.1039/d2cs00206j

[53] O. S. Kamble , R. Chatterjee , R. Dandela , Arkivoc 2022, 5, 270–281.

[54] P. Sureshbabu , S. Azeez , P. Chaudhary , J. Kandasamy , Org. Biomol. Chem. 2019, 17, 845–850. 30627716 10.1039/c8ob03010c

[55] F. Nasiri , J. Mokhtari , S. Taheri , Z. Mirjafary , Tetrahedron Lett. 2023, 118, 154392.

[56] R. Fu , Y. Yang , Z. Chen , W. Lai , Y. Ma , Q. Wang , R. Yuan , Tetrahedron 2014, 70, 9492–9499.

[57] V. Singh , K. Rajput , A. Mishra , S. Singh , V. Srivastava , Chem. Commun. 2023, 59, 14009–14012. 10.1039/d3cc04128j 37941417

[58] a) S. N. Rao , D. C. Mohan , S. Adimurthy , Org. Lett. 2013, 15, 1496–1499; 23473076 10.1021/ol4002625

[59] Q. Tian , Z. Gan , X. Wang , D. Li , W. Luo , H. Wang , Z. Dai , J. Yuan , Molecules 2018, 23, 2234. 30200533 10.3390/molecules23092234 PMC6225136

[60] F. V. Singh , S. E. Shetgaonkar , M. Krishnan , T. Wirth , Chem. Soc. Rev. 2022, 51, 8102–8139. 36063409 10.1039/d2cs00206j

[61] R. Vanjari , B. K. Allam , K. N. Singh , R. S. C. Adv., 2013, 3, 1691–1694.

[62] C. Larrivée‐Aboussafy , B. P. Jones , K. E. Price , M. A. Hardink , R. W. McLaughlin , B. M. Lillie , J. M. Hawkins , R. Vaidyanathan , Org. Lett. 2010, 12, 324–327. 20014771 10.1021/ol9026599

[63] T. M. Dine , D. Evans , J. Rouden , J. Blanchet , Chem. ‐ Eur. J. 2016, 22, 5894–5898. 26946179 10.1002/chem.201600234

[64] J. Ma , J. Zhang , X. Zhou , J. Wang , H. Gong , J. Iran. Chem. Soc. 2018, 15, 2851–2860.

[65] S. Bhattacharya , P. Ghosh , B. Basu , Tetrahedron Lett. 2018, 59, 899–903.

[66] K. P. Patel , E. M. Gayakwad , V. V. Patil , G. S. Shankarling , Adv. Synth. Catal. 2019, 361, 2107–2116.

[67] K. P. Patel , S. S. Kamble , D. R. Boraste , G. S. Shankarling , Environ. Chem. Lett. 2020, 18, 1731–1735.

[68] R. Dalpozzo , G. Bartoli , L. Sambri , P. Melchiorre , Chem. Rev. 2010, 110, 3501–3551. 20235581 10.1021/cr9003488

[69] D. Joseph , M. S. Park , S. Lee , Org. Biomol. Chem. 2021, 19, 6227–6232. 34225358 10.1039/d1ob00967b

[70] S. Rasheed , D. N. Rao , A. S. Reddy , R. Shankar , P. Das , R. S. C. Adv , 2015, 5, 10567–10574.

[71] W. Guo , J. Huang , H. Wu , T. Liu , Z. Luo , J. Jian , Z. Zeng , Org. Chem. Front. 2018, 5, 2950–2954.

[72] J. Chen , Y. Xia , S. Lee , Org. Lett. 2020, 22, 3504–3508. 32298591 10.1021/acs.orglett.0c00958

[73] a) C. L. Allen , B. N. Atkinson , J. M. Williams , Angew. Chem., Int. Ed. 2012, 51, 1383–1386; 10.1002/anie.201107348 22213128

[74] T. B. Nguyen , J. Sorres , M. Q. Tran , L. Ermolenko , A. Al‐Mourabit , Org. Lett. 2012, 14, 3202–3205. 22676810 10.1021/ol301308c

[75] A. S. Mali , K. Indalkar , G. U. Chaturbhuj , Org. Prep. Proced. Int. 2021, 53, 369–378.

[76] E. Eidi , M. Z. Kassaee , Z. Nasresfahani , J. Nanopart. Res. 2018, 20, 1–9.

[77] M. M. Rahman , G. Li , M. Szostak , J. Org. Chem. 2019, 84, 12091–12100. 31430149 10.1021/acs.joc.9b02013

[78] O. Verho , M. Pourghasemi Lati , M. Oschmann , J. Org. Chem. 2018, 83, 4464–4476. 29578345 10.1021/acs.joc.8b00174

[79] V. A. Fiore , G. Maas , Tetrahedron 2019, 75, 3586–3595.

[80] S. Shi , R. Lalancette , R. Szostak , M. Szostak , Org. Lett. 2019, 21, 1253–1257. 30768275 10.1021/acs.orglett.8b03901

[81] I. A. Rajan , S. Rajendran , New J. Chem. 2023, 47, 10480–10483.

[82] F. O. Bensalah , A. Bil , K. Wittine , S. Bellahouel , D. Lesur , D. Markovic , S. Laclef , Org. Biomol. Chem. 2019, 17, 9425–9429. 31651020 10.1039/c9ob02096a

[83] T. Lebleu , H. Kotsuki , J. Maddaluno , J. Legros , Tetrahedron Lett. 2014, 55, 362–364.

[84] J. Yin , J. Zhang , C. Cai , G. J. Deng , H. Gong , Org. Lett. 2018, 21, 387–392. 30588817 10.1021/acs.orglett.8b03542

